# Maternal and Fetal Outcome of COVID-19 Infection among Pregnant Women

**DOI:** 10.3390/medicina60101676

**Published:** 2024-10-12

**Authors:** Eman M. Khalil, Yasmin M. Madney, Mahmoud Hassan, Alzhraa M. Fahmy, Saud O. Alshammari, Qamar A. Alshammari, Heba A. Abou-Taleb, Ahmed A. Taha, Marwa O. Elgendy, Hamada A. A. Ali

**Affiliations:** 1 Department of Obstetrics and Gynecology, Faculty of Medicine, Beni-Suef University, Beni-Suef 62521, Egypt; emaanmoustafaa@gmail.com (E.M.K.); ahmedabdulkhaliq@med.bsu.edu.eg (A.A.T.); hamadaashry2010@yahoo.com (H.A.A.A.); 2 Department of Clinical Pharmacy, Faculty of Pharmacy, Beni-Suef University, Beni-Suef 62521, Egypt; yasminmohamed5050@yahoo.com; 3 Department of Internal Medicine, Faculty of Medicine, Beni-Suef University, Beni-Suef 62521, Egypt; mahmoudhassanahmady@gmail.com; 4 Tropical Medicine and Infectious Diseases Department, Faculty of Medicine, Beni-Suef University, Beni-Suef 62521, Egypt; alzhraamohamed@med.bsu.edu.eg; 5 Department of Pharmacognosy and Alternative Medicine, Faculty of Pharmacy, Northern Border University, Rafha 76321, Saudi Arabia; saud.o.alshammari@nbu.edu.sa; 6 Department of Pharmacology and Toxicology, Faculty of Pharmacy, Northern Border University, Rafha 76321, Saudi Arabia; qamar.alshammari@nbu.edu.sa; 7 Department of Pharmaceutics and Industrial Pharmacy, Faculty of Pharmacy, Merit University (MUE), Sohag 82755, Egypt; heba.ahmed@merit.edu.eg; 8 Department of Clinical Pharmacy, Beni-Suef University Hospitals, Faculty of Medicine, Beni-Suef University, Beni-Suef 62521, Egypt; 9 Department of Clinical Pharmacy, Faculty of Pharmacy, Nahda University (NUB), Beni Suef 62764, Egypt

**Keywords:** pregnancy, COVID-19, three trimesters, fetus, SARS-CoV-2, post-COVID syndrome

## Abstract

*Background and Objectives*: Pregnant women face an increased risk of experiencing negative consequences due to COVID-19 infection. Our study aimed to identify outcomes for both mothers and fetuses associated with COVID-19 during each trimester, as well as to identify post-COVID symptoms in this population. *Materials and Methods*: Among the total population, 14 females were infected during the first trimester, 25 during the second, and 66 during the third trimester. Weekly follow-ups were conducted until delivery. Seventy-five females (71.4%; 95% CI:26.9–115.9%) were admitted to the hospital secondary to COVID-19 infection. Maternal hospitalization was independently associated with COVID-19 severity (adjusted odds ratio (aOR) = 3.9; 95% CI: 1.6–9.2 at *p* = 0.002 relative to the reference group (mild infection)) and the presence of dyspnea at initial assessment (aOR = 6.9; 95% CI: 1.7–28.2 at *p* = 0.007 relative to nondyspneic patients). *Results*: The duration of hospitalization (mean ± SD) was higher in the third trimester than the first and second trimesters (10.1 ± 0.8 vs. 4.0 ± 1.2 days and 10.1 ± 0.8 vs. 6.2 ± 1.4 days, respectively, at *p <* 0.05). The number of maternal deaths in the third trimester was higher than in the first and second trimesters (16 (24.2%) vs. no deaths and 16 (24.2%) vs. 1 (4%) deaths, respectively, at *p <* 0.05). In terms of fetal outcomes, a good fetal condition was more likely if the mother was infected during the first trimester (92.9%) than the second (80%) or third trimesters (66.7%), but the difference was not significant. The percentage of preterm deliveries was insignificantly higher in the second trimester (16%) than the first (7.1%) and third (4.5%) trimesters. *Conclusions:* The most common post-COVID symptoms included persistent loss of smell, dry eyes, post-partum depression, knee pain, and myalgia. Post-COVID symptoms were more prevalent in patients infected during the third trimester. The adverse outcomes of COVID-19 infection for both mother and fetus were more severe in cases where the infection occurred during the third trimester compared to the second and first trimesters. Therefore, it is crucial to adhere to precautionary measures against COVID-19, prioritize vaccination, and provide comprehensive care for pregnant mothers.

## 1. Introduction

The connection between COVID-19 and pregnancy was initially dismissed at the beginning of the pandemic [1,2]. However, recent research has shown that women who are pregnant or have just delivered babies are more vulnerable to the negative consequences of severe acute respiratory syndrome coronavirus 2 (SARS-CoV-2) infection [3,4]. Pregnant women with COVID-19 infections faced an elevated risk of being hospitalized, intensive care unit (ICU) admission, mechanical ventilation, and premature birth, according to a previous study [5,6].

Changes in the immune system and physiology occur during pregnancy, raising the risk of COVID-19 infection in both the mother and the fetus [7]. From the beginning of the pandemic, more cases of maternal morbidity and mortality linked to the virus have been reported [8]. COVID-19 has the potential to cause obstetric complications such as preterm labor, miscarriage, pre-eclampsia, and fetal distress [9].

According to a study conducted in the United States in March 2020 on 43 hospitalized pregnant women, 30% of them needed to be admitted to the intensive care unit, 14% needed mechanical ventilation, and one of them died from COVID-19 [10]. In 2020, the COVID-19-related death rate amongst pregnant and postpartum women in Brazil achieved a milestone record of 12.7%, representing the greatest rate in the world [11].

Studies have revealed that in low- and middle-income developing countries, there are negative factors that increase the risk of COVID-19 infection among mothers. These factors include the insufficient distribution of human and physical resources, insufficient protective measures, increased rates of birth, and ineffective prenatal care [11,12,13].

As seen previously with SARS, pregnant women experienced higher rates of fetal loss, intrauterine growth restriction (IUGR), preterm delivery, and maternal death. A case–control study on SARS patients reported that pregnant patients had a worse prognosis and a worse clinical outcome than non-pregnant patients [14]. Pregnant women who have contracted MERS are also at increased risk of experiencing premature delivery, perinatal morbidity, and high maternal death rates [14].

While some investigations have examined the effects of COVID-19 throughout pregnancy, the majority of these studies were small case series from China that concentrated on third-trimester pregnant women [15]. Therefore, in order to direct treatment and avoid complications for pregnant women with COVID-19, more information is needed regarding the effects of the virus during the various trimesters of pregnancy. An increasingly recognized COVID-19 infection complication is post-COVID-19 syndrome, also referred to as long COVID [16,17]. Pregnancy makes it especially difficult to diagnose and treat post-COVID-19 syndrome. The most common symptoms of post-COVID-19 syndrome are shortness of breath, fatigue, and cognitive diminishing or ‘brain fog’ similar to symptoms often reported by women during normal pregnancy [18].

This study examined maternal and fetal outcomes associated with COVID-19 infection in the three trimesters and identified post-COVID syndrome in them. As far as we are aware, this is the biggest Egyptian study to date.

## 2. Materials and Methods

### 2.1. The Study Design

A cohort study was conducted between December 2021 and May 2022, involving 105 pregnant women who presented with COVID-19 infection at the obstetrics and gynecology clinic. The study protocol received approval from the Research Ethical Committee of Beni-Suef University and was implemented at Beni-Suef University Hospital in adherence to the Helsinki Declaration, and written informed consent was obtained from all participants. Among the total patients, 14 were in the first trimester, 25 in the second trimester, and 66 in the third trimester. Laboratory confirmation of SARS-CoV-2 infection was achieved using reverse transcription polymerase chain reaction (RT-PCR) for all patients. Treatment for COVID-19 followed the guidelines of both the World Health Organization (WHO) and the Egyptian protocol.

Pregnant women aged 18 years or older who were positive for COVID-19 as confirmed by RT-PCR test were included in the study, while women with chronic medical disorders or who were less than 18 years old were excluded from the study.

### 2.2. Sampling Techniques

The analysis involved examining data collected from pregnant women following the confirmation of their COVID-19 infection. The samples obtained for analysis included blood and nasal swabs. Fetal monitoring through ultrasound and Doppler was conducted during pregnancy, and the Apgar score was assessed after delivery.

#### Data Collection

Information was gathered from the patients, encompassing the following aspects:

- The demographic and clinical parameters of the participants;
- Evaluation of the impact of COVID-19 infection during different trimesters on mothers and the identification of post-COVID symptoms;
- Assessment of the effects of COVID-19 infection on the fetus during the different trimesters.

The clinical parameters were specifically compiled to identify maternal and fetal outcomes associated with COVID-19 infection across the three trimesters and to detect post-COVID syndrome.

### 2.3. Statistical Analysis

Descriptive analysis was performed, with continuous variables presented as the mean ± SD and categorical variables expressed as the number of cases and percentage values. The normal distribution of continuous variables was assessed using the Kolmogorov–Smirnov and Shapiro–Wilk tests. Differences in baseline demographic data and clinical parameters among the three trimesters were evaluated using the Kruskal–Wallis H test, followed by the Mann–Whitney U test for continuous variables. The chi-square test was employed to determine differences in categorical data among the different trimesters. Statistical significance was defined as *p* < 0.05. Multiple logistic regression models were used to examine the risk factors for important study outcomes (maternal hospitalization and complications). Only risk factors with a *p* value < 0.20 in the univariable analysis were included in the multivariable analyses for each binary outcome. Logistic regression with backward stepwise selection was used to choose risk factors for each multivariable model. A significance level of 0.20 was required to allow a risk factor into the model, and a significance level of 0.20 was required for a risk factor to stay in the model. All statistical analyses were carried out using SPSS v17.0 (SPSS, Chicago, IL, USA).

## 3. Results

### 3.1. Participants’ Demographics and Clinical Parameters

A total of 105 pregnant women were enrolled in the study, with an average age of 27.9 ± 6.5 years (mean ± SD). The duration of pregnancy, measured in weeks, averaged 28.4 ± 9.9. The mean Total Leukocyte Count (TLC) and Hemoglobin were 12.2 ± 6.9 and 10.1 ± 1.4, respectively. A majority of the participants (88.6%) experienced lymphopenia. The mean Serum Ferritin, C-Reactive Protein (CRP), and serum creatinine were 360.5 ± 310.4, 89.3 ± 103.9, and 1.1 ± 0.4, respectively. A significant portion of the participants (76.2%) had elevated D-dimer levels. Among all participants, 13.3% were diagnosed with gestational diabetes. The majority of participants reported suffering from moderate (40%) and mild (31.4%) COVID-19 infections. Regarding COVID-19 symptoms, fever (98.1%), cough (78.1%), and dyspnea (46.7%) were the most commonly reported.

Detailed demographic and clinical parameters and the symptoms of the participants are presented in Table 1 and Table 2.

### 3.2. Effect of COVID-19 Infection at Different Trimesters on Mother

Admission rates to the hospital were 50%, 52%, and 83.3% for the first, second, and third trimesters, respectively. The average admission durations (mean ± SD) were 4 ± 1.2, 6.2 ± 1.4, and 10.1 ± 0.8 for the first, second, and third trimesters, respectively.

The need for mechanical ventilation was 0%, 12%, and 27.3% for the first, second, and third trimesters, respectively. However, the rates of patients requiring supplemental oxygen therapy were 42.9%, 48%, and 66.7% for the first, second, and third trimesters, respectively. Respiratory distress complications were prevalent in 35.7%, 24%, and 65.2% of patients for the first, second, and third trimesters, respectively.

The fetal loss occurred in 7.1% of first-trimester infected women, 16% of second-trimester infected women, and 3% of third-trimester infected women. Complications such as accidental hemorrhage, vaginal hematoma, retroplacental hematoma, broad ligament hematoma, endometriosis, convulsions, wound infection, and pulmonary edema were observed in third-trimester infected women. Pregnancy continued to full term in 92.9%, 80%, and 15.2% of patients in the first, second, and third trimesters, respectively.

Regarding post-COVID syndrome, persistent loss of smell was found in 12.4% of participants, while dry eyes and sexual dysfunction were found in 6.7% of all participants. Post-partum depression was observed in 10.6% of third-trimester infected women, and myalgia was found in 5.7% of all participants. Nearly, a quarter of the third trimester infected women died.

The effect of COVID-19 infection at different trimesters on the mother is presented in Table 3.

### 3.3. Effect of COVID-19 Infection at Different Trimesters on the Fetus

The Umbilical Artery Resistance Index (RI) values (mean ± SD) were 0.4 ± 0.1, 0.5 ± 0.03, and 0.7 ± 0.02 for the first, second, and third trimesters, respectively. In the third trimester, 1.5% of cases experienced intrauterine growth restriction (IUGR), while 15.2% had intrauterine fetal demise (IUFD). After birth, 4% of second-trimester cases and 13.6% of third-trimester cases resulted in infant mortality. The overall well-being of the fetus was observed in 92.9%, 80%, and 66.7% for the first, second, and third trimesters, respectively.

The effect of COVID infection on the fetus at different trimesters is presented in Table 4.

### 3.4. Univariable Logistic Regression and Multiple Logistic Regression Analysis

Table 5 illustrates univariable logistic regression results to predict the most significant maternal outcomes. Maternal hospitalization was independently associated with COVID-19 severity (adjusted odds ratio (aOR) = 3.9; 95% CI: 1.6–9.2 at *p* = 0.002 relative to the reference group (mild infection)) and presence of dyspnea at initial assessment (aOR = 6.9; 95% CI: 1.7–28.2 at *p* = 0.007 relative to nondyspneic patients).

The risk of developing respiratory distress complications in pregnant females can be mainly determined by the presence of Co-RADS infiltration > 50% in chest imaging (aOR = 32.8; 95% CI: 5.9–180.3 at *p* < 0.001) relative to the reference level (without Co-RADS infiltration > 50%) as illustrated in Table 6.

## 4. Discussion

According to the study’s findings, pregnant women who contract COVID-19 during their pregnancy may have a higher risk of death due to worsening maternal and fetal outcomes. This aligns with previous studies [19,20].

In this research, advanced age and comorbidities such as hypertension, chronic diabetes, bronchial asthma, hyperparathyroidism, rheumatoid arthritis (RA), and systemic lupus erythematosus (SLE) were associated with worsened outcomes, particularly in participants infected during the third trimester. Previous studies have also connected the presence of COVID-19 to increased risks of admissions to hospitals [21,22], respiratory problems, and mortality [23]. These factors include age, asthma, obesity, and pregnancy [20]. Prior research has shown that women with comorbidities and associated risk factors who were pregnant or recently gave birth had increased odds of experiencing negative outcomes from COVID-19 [13,24]. Pregnant women infected in the second and third trimesters faced an increased risk of gestational diabetes (GDM), as reported in previous studies [25].

A significant portion of the individuals who contracted the disease during the third trimester developed a severe case of COVID-19 infection. Severe infection was reported in the third trimester more than the second and in the second more than in the first. Severe illness was more common in later pregnancy, as reported by the UK Obstetric Surveillance System (UKOSS) study, where most pregnant women were hospitalized in the third trimester or peripartum [26]. Adverse maternal and neonatal outcomes, such as preterm delivery, neonatal infection, low neonatal birth weight, and/or admission to the neonatal intensive care unit (NICU), have been linked to pregnancies with severe forms of COVID-19 [27,28]. In general, patients with severe clinical manifestation had much higher incidence rates of the aforementioned outcomes than patients with a straightforward course of the disease. Similarly, a number of studies found that pregnant women with severe COVID-19 had a higher chance of unfavorable obstetric and neonatal outcomes [29].

Third-trimester participants had worsening chest conditions, which may raise the possibility of preterm delivery [30].

Compared to those infected in the second and first trimesters, pregnant women required longer hospital stays in the third trimester due to their heightened susceptibility to emergencies and infectious diseases [29] pregnant women who are infected have a higher risk of developing severe COVID-19 infections, with a significant proportion requiring mechanical ventilation and intensive care [31,32]. Furthermore, according to the Centers of Disease Control and Prevention (CDC), hospitalization rates for pregnant women with SARS-CoV-2 (COVID-19) infection were higher than those for non-pregnant individuals at the same age (31.5% vs. 5.8%) [33]. According to the UKOSS study, where the majority of pregnant women were admitted to hospital in the third trimester or postpartum, severe illness seems to be more prevalent in later pregnancy [26].

This study clearly demonstrated the deteriorating state of the infected cases during the third trimester, as over 25% required mechanical ventilation and two thirds required oxygen therapy sublimentation. Moreover, the most prevalent complication in the study cases was respiratory distress, especially in third-trimester cases. The immunologic and physiological changes that occur during pregnancy, such as reduced respiratory capacity, elevated oxygen demand, aspiration risk, and diminished maternal tolerance to hypoxia, can be linked to the severity of maternal disease and the obstetric outcomes [29,34]. According to a previous study, intensive care unit (ICU) admission, hospitalization, mechanical ventilation, and premature birth are common outcomes for pregnant women with COVID-19 infections [5]. In March 2020, an American study involving forty-three hospitalized pregnant women found that fourteen percent needed mechanical ventilation and thirty percent needed admission to an intensive care unit (ICU) [10].

In the second and third trimesters, miscarriage, preterm labor, and endometriosis were common. There was a significant relationship between severe COVID-19 and iatrogenic preterm delivery, particularly in the third trimester [35]. Similarly, a systematic review involving 31,016 pregnant women from 62 studies confirmed our findings showing an around twofold increase in preterm labor among pregnancies with severe COVID-19 symptoms compared to the control [36]. These outcomes may be connected to the mother’s pneumonia during the COVID-19 course, which is the primary cause of pregnancy complications such as premature labor, placental abruption, and potentially fatal outcomes for the mother or the fetus [37].

Complications such as vaginal hematoma, retroplacental hematoma, broad ligament hematoma, postpartum hemorrhage, and wound infection were more common in third-trimester cases. Factors such as chronic hematological disease and immunosuppression are associated with COVID-19 infection [20,38,39].

Many COVID-19 survivors reported that their symptoms persisted even after recovery from the infection. “Post-COVID Condition” or “Long-COVID” have been terms used to describe this condition [40]. The study found that persistent loss of smell, dry eyes, knee pain, post-partum depression, myalgia, persistent headache, backache, insomnia, and persistent fever were among the post-COVID symptoms that some patients reported experiencing. The most common post-COVID symptoms in pregnant women, according to a prior study, were exhaustion, hair loss, and difficulties concentration [40,41,42]. The findings of this earlier study imply that there is little difference between reports from general population studies and the manner of presentation of long-term sequelae of SARS-CoV-2 infection in pregnant women [40].

A notable proportion of participants in the third trimester experienced mortality, consistent with findings from previous pandemics and seasonal influenza, emphasizing the higher risk faced by pregnant women for morbidity and mortality linked to the infection [43]. This is due to changes in the cardiorespiratory and immune systems during pregnancy which increase susceptibility to severe infections and hypoxic compromise [44].

In fact, there is a considerable death rate among pregnant populations related to infections. One of the main causes of maternal deaths globally is viral pneumonia [45], which carries an extra risk for SARS-CoV-2-positive pregnant women because it enters cells through the angiotensin-converting enzyme receptor 2 (ACE2), which increases the expression of the virus during normal pregnancy [43]. However, empirical validation of this elevated risk is still pending, and there is currently no conclusive evidence regarding the virus’s intrauterine vertical transmission [45,46].

In terms of the effects on the fetus, the third-trimester cases in this study exhibited worse fetal conditions than those in the second and first trimesters, with 13.6% of fetuses from third-trimester cases experiencing mortality after birth. Previous studies have reported the potential risks of placental infection or vertical transmission, which can result in a variety of complications, such as preterm birth abortion, fetal distress, cesarean section, premature delivery, NICU admission, growth restrictions, and early-onset neonatal sepsis [15,47,48].

In addition to intrauterine vertical transmission, viral infection can also be acquired during the fetus’s passage through the birth canal, during postpartum breastfeeding, through skin contact, or by the baby inhaling respiratory droplets from mother or any nearby person when they cough or sneeze. This information is crucial for determining whether a neonatal infection was acquired prior to or after delivery [37]. Also, a previous study reported that the effect of SARS-CoV-2 infection on the fetus in the first or second trimester or in patients with moderate to severe infection is unknown but, in the third trimester, may cause preterm birth, intrauterine growth restriction, intrauterine death, and neonatal death [49].

The Umbilical Artery Resistance Index (RI), is used in the surveillance of fetal well-being in pregnancy. An RI of >0.58 was defined as abnormal and an RI of > or = 0.7 was defined as very abnormal [50]. The determination of the Umbilical Artery Resistance Index (RI) in this study revealed very abnormal RIs in third-trimester cases, indicating placental insufficiency and potential intrauterine growth restriction (IUGR) or suspected pre-eclampsia [50].

Our study has several potential limitations. First, it is a single-center study, and the sample size may be relatively small, which could limit the generalizability of the findings to a wider population. Additionally, the number of pregnant women infected with COVID-19 during the third trimester was significantly higher than in the second and first trimesters. There are also potential limitations regarding the availability and accuracy of historical data, which may have led to some errors in documenting events and outcomes. Despite these limitations, our findings could hold substantial clinical importance. We stress the need to prioritize preventive and therapeutic strategies for pregnant women in their third trimester when addressing COVID-19.

## 5. Conclusions

Pregnant individuals in their third trimester exhibited a higher likelihood of developing severe COVID-19 infections and were more susceptible to hospital admission compared to those in the second or first trimester. The duration of hospitalization, the necessity for mechanical ventilation, and the risk of respiratory distress were all higher in the third trimester than in the second or first trimester. Conversely, the risk of abortion and preterm labor was greater in the second trimester than in the first or third trimester. Maternal death risk was identified in nearly a quarter of patients infected in the third trimester. When the mother contracted COVID-19 in the first trimester compared to the second or third, there was typically a favorable fetal condition observed. The risks of intrauterine growth restriction (IUGR), intrauterine fetal demise (IUFD), and fetal death after birth were elevated in third-trimester cases. The most prevalent post-COVID symptoms included persistent loss of smell, dry eyes, post-partum depression, knee pain, and myalgia, with a higher incidence in patients infected during the third trimester. The adverse outcomes of COVID-19 infection on both the mother and fetus were more severe in those infected during the third trimester compared to those infected during the second or first trimester. Therefore, it is crucial to adhere to precautionary measures against COVID-19, promote vaccination, and provide comprehensive care for pregnant mothers.

## Figures and Tables

**Table 1:** Baseline demographic and clinical parameters are represented as mean ±SD or *n* (%).

	Total Population (*n* = 105)	First Trimester (*n* = 14)	Second Trimester (*n* = 25)	Third Trimester (*n* = 66)	*p*-Value		
					1st vs. 2nd Trimester	2nd vs. 3rd Trimester	1st vs. 3rd Trimester
Age (years)	27.9 ± 6.5	23.7 ± 1.5	24.2 ± 1.1	30.2 ± 0.7	0.837	<0.001	0.001
Duration of pregnancy (weeks)	28.4 ± 9.9	10.3 ± 0.7	20.9 ± 0.8	35.1 ± 0.4	<0.001	<0.001	<0.001
TLC (X109/L)	12.2 ± 6.9	10.6± 1.5	11.5 ± 0.9	12.8 ± 0.9	0.461	0.772	0.329
Lymphocyte count					0.916	0.058	0.086
Normal level	11 (10.5%)	3 (21.4%)	5 (20%)	3 (4.5%)			
Low level	93 (88.6%)	11 (78.6%)	20 (80%)	62 (94%)			
High level	1 (0.9%)	-	-	1 (1.5%)			
Hemoglobin (mg/dL)	10.1 ± 1.4	10.9 ± 0.3	10.5 ± 0.3	9.8 ± 0.2	0.359	0.016	0.006
Serum ferritin (ng/mL)	360.5 ± 310.4	198.7 ± 56.3	399.9 ± 64.7	379.8 ± 38.9	0.101	0.845	0.057
Serum creatinine (mg/dL)	1.1 ± 0.4	0.95 ± 0.08	1.02 ± 0.05	1.16 ± 0.06	0.292	0.528	0.142
CRP (mg/L)	89.3 ± 103.9	116.1 ± 45.3	56.6 ± 14.9	95.9 ± 11.6	0.385	0.004	0.284
D-dimer level					0.440	0.094	0.828
Normal	2 (1.9%)	-	1 (4%)	1 (1.5%)			
Low	23 (21.9%)	3 (21.4%)	9 (36%	11 (16.7%)			
High	80 (76.2%)	11 (78.6%)	15 (60%)	54 (81.8%)			
SGPT					0.277	0.063	0.032
Normal	86 (81.9%)	14 (100%)	23 (92%)	49 (74.2%)			
High	19 (18.1%)	-	2 (8%)	17 (25.8%)			
Comorbidities					0.278	0.756	0.779
Hypertension	10 (9.5%)	1 (7.1%)	2 (8%)	7 (10.6%)			
Chronic diabetes	10 (9.5%)	-	4 (16%)	6 (9.1%)			
Bronchial asthma	2 (1.9%)	-	-	2 (3%)			
Hyper-parathyroidism	2 (1.9%)	-	-	2 (3%)			
RA	1 (0.95%)	-	-	1 (1.5%)			
SLE	3 (2.9%)	-	-	3 (3%)			
Gestational diabetes	14 (13.3%)	1 (7.1%)	6 (24%)	7 (10.6%)	0.188	0.103	0.695
Other co-existing infections					-	0.735	0.0889
UTI	1 (0.95%)	-	-	1 (1.5%)			
Skin infection	2 (1.9%)	-	-	2 (3%)			
CNS infection	1 (0.95%)	-	-	1 (1.5%)			
Encephalitis	1 (0.95%)	-	-	1 (1.5%)			
Severity of COVID infection							
Mild	33 (31.4%)	8 (57.1%)	10 (40%)	15 (22.7%)	0.564	0.108	0.031
Moderate	42 (40%)	4 (28.6%)	11 (44%)	27 (40.9%)			0.389
Severe	30 (28.6%)	2 (14.3%)	4 (16%)	24 (36.4%)			0.109
Chest imaging							
Co-RADS infiltration > 50 %	36 (34.3%)	0/14 (0%)	4 (16%)	32 (48%)	0.274	0.007	0.001

Abbreviations: TLC, total leukocyte count; CRP, C-Reactive Protein; SGPT, serum glutamate pyruvate transaminase; RA, rheumatoid arthritis; SLE, systemic lupus erythematosus; UTI: urinary tract infection; Co-RADS: COVID19 reporting and data system.

**Table 2:** COVID-19 symptoms are represented as mean ±SD or *n* (%).

	Total Population (*n* = 105)	First Trimester (*n* = 14)	Second Trimester (*n* = 25)	Third Trimester (*n* = 66)	*p*-Value		
					1st vs. 2nd Trimester	2nd vs. 3rdTrimester	1st vs. 3rd Trimester
Symptoms					0.076		
Fever	103 (98.1%, 95% CL: 46–149%)	13 (92.9%)	24 (96%)	66 (100%)		0.102	0.029
Cough	82 (78.1%, 95% Cl: 31–125.2%)	10 (71.4%)	16 (64%)	56 (84.8%)		0.029	0.23
Dyspnea	49 (46.7%, 95% CL: 13.7–79.8%)	2 (14.3%)	8 (32%)	39 (59.1%)		0.021	0.002
Loss of smell	5 (4.8%, 95% CL: 1.8–11.3)	1 (7.1%)	2 (8%)	2 (3%)		0.302	0.462
Malaise	13 (12.4%, 95% CL: 0.2–25%)	2 (14.3%)	2 (8%)	9 (13.6%)		0.462	0.949
Headache	6 (5.7%, 95% CL: 1.7–13.1%)	2 (14.3%)	2 (8%)	2 (3%)		0.302	0.079
Muscle pain	2 (1.9%, 95% CL: 0.2–5.6%)	-	2 (8%)	-		0.020	-
Convulsions	1 (0.95%, 95% CL: 0.47–3.3)	-	-	1 (15.1%)		0.536	0.643

**Table 3:** Effect of COVID infection at different trimesters on mothers represented as *n* (%).

	Total Population (*n* = 105)	First Trimester (*n* = 14)	Second Trimester (*n* = 25)	Third Trimester (*n* = 66)	*p*-Value		
					1st vs. 2nd Trimester	2nd vs. 3rd Trimester	1st vs. 3rd Trimester
Hospitalization	75 (71.4%)	7 (50%)	13 (52%)	55 (83.3%)	0.905	0.002	0.007
Admission period	8.4 ± 6.9	4 ± 1.2	6.2 ± 1.4	10.1 ± 0.8	0.369	0.024	0.002
Supplemental oxygen therapy	62 (59%)	6 (42.9%)	12 (48%)	44 (66.7%)	0.757	0.102	0.095
Mechanical ventilation	21 (20%)	-	3 (12%)	18 (27.3%)	0.177	0.123	0.026
Complications					0.435		0.094
Respiratory distress	55 (52.4%)	5 (35.7%)	6 (24%)	43 (65.2%)		0.001	
CNS complications	1 (0.95%)	-	-	1 (1.5%)		0.536	
Number of miscarriages or fetal losses	7 (6.7%)	1 (7.1%)	4 (16%)	2 (3%)	0.427	0.026	0.462
Number of preterm labors	8 (7.6%)	1 (7.1%)	4 (16%)	3 (4.5%)	0.427	0.067	0.685
Obstetric complications and accidental finding						0.536	0.443
Chorioamnionitis and ROM	1 (0.95%)	-	1 (4%)	-	0.448		
Accidental hemorrhage	1 (0.95%)	-	-	1 (1.5%)	-		
Vaginal hematoma	1 (0.95%)	-	-	1 (1.5%)	-		
Retroplacental hematoma	8 (7.6%)	1 (7.1%)	1 (4%)	6 (9.1%)	0.669		
Broad ligament hematoma	1 (0.95%)	-	-	1 (1.5%)	-		
Endometriosis	3 (2.9%)	-	1 (4%)	2 (3%)	0.448		
Post-partum hemorrhage	17 (16.2%)	4 (28.6)	1 (4%)	12 (18.1%)	0.028		
Convulsions	1 (0.95%)	-	-	1 (1.5%)	-		
Wound infection	4 (3.8%)	-	1 (4%)	3 (4.5%)	0.448		
Pulmonary edema	1 (0.95%)	-	-	1 (1.5%)	-		
Delivery					0.682		
Continued pregnancy	43 (41%)	13 (92.9%)	20 (80%)	10 (15.2%)		<0.001	<0.001
Vaginal delivery	15 (14.3%)	-	1 (4%)	14 (21.2%)		0.048	0.058
Cesarean section	38 (36.2%)	-	-	38 (57.6%)		<0.001	<0.001
Hysterotomy	2 (1.9%)	-	1 (4%)	1 (1.5%)		0.470	0.643
Post-COVID sequalae					0.699		
Persistent loss of smell	13 (12.4%)	2 (14.3%)	2 (8%)	9 (13.6%)		0.462	0.949
Dry eyes	7 (6.7%)	1 (7.1%)	1 (4%)	5 (7.6)		0.540	0.955
Sexual dysfunction	7 (6.7%)	1 (7.1%)	4 (16%)	2 (3%)		0.026	0.462
Post-partum depression	8 (7.6%)	-	1 (4%)	7 (10.6%)		0.321	0.202
Knee pain	4 (3.8%)	2 (14.3%)	2 (8%)	-		0.020	0.002
Myalgia	6 (5.7%)	2 (14.3%)	1 (4%)	3 (4.5%)		0.910	0.171
Backache	1 (0.95%)	-	1 (4%)	-		0.102	-
Persistent headache	2 (1.9%)	-	2 (8%)	-		0.020	-
Insomnia	1 (0.95%)	1 (7.1%)	-	-		-	0.029
Persistent fever	2 (1.9%)	-	-	2 (3%)		0.379	0.509
Mother death	17 (16.2%)	-	1 (4%)	16 (24.2%)		0.027	0.039

Abbreviations: ROM, spontaneous rupture of membranes.

**Table 4:** Effects of COVID infection on the fetus at different trimesters represented as mean ±SD or *n* (%).

	Total Population (*n* = 105)	First Trimester (*n* = 14)	Second Trimester (*n* = 25)	Third Trimester (*n* = 66)	*p*-Value		
					1st vs. 2nd Trimester	2nd vs. 3rd Trimester	1st vs. 3rd Trimester
Umbilical artery RI	0.6 ± 0.2	0.4 ± 0.1	0.5 ± 0.03	0.7 ± 0.02	0.310	<0.001	0.001
Fetal condition					0.472	0.101	0.209
IUGR	1 (0.95%)	-	-	1 (1.5%)			
IUFD	10 (9.5%)	-	-	10 (15.2%)			
Good	77 (73.3%)	13 (92.9)	20 (80%)	44 (66.7%)			
Death after birth	10 (9.5%)	-	1 (4%)	9 (13.6%)			

Abbreviations: RI, resistance index.

**Table 5:** Univariable logistic regression to predict maternal outcomes during COVID-19.

Outcome	Risk Factor	Coefficient	SE	*p*-Value	Odds Ratio (OR)	95% CI for aOR	
						Lower Bound	Upper Bound
Maternal hospitalization ^#^	Severity of COVID infection *	1.815	0.402	<0.001	6.1	2.8	13.5
	Dyspnea ^#^	2.659	0.653	<0.001	14.3	3.9	51.4
	Lymphocyte count ^&^	1.701	0.663	0.01	5.5	1.5	20.1
Respiratory distress complication ^#^	Age ^$^	0.109	0.035	0.002	1.12	1.04	1.2
	Cough ^#^	1.121	0.505	0.026	3.1	1.1	8.3
	Dyspnea ^#^	1.956	0.441	<0.001	7.1	2.9	16.8
	Chest imaging (Co-RADS infiltration > 50%) ^#^	3.729	0.774	<0.001	41.7	9.1	190

“Coefficient” is the estimated regression coefficient. Abbreviations: Co-RADS: COVID-19 reporting and data system. ^#^: the reference category is no. *: the reference category for the severity of COVID infection is a mild state; ^&^: the reference category for lymphocyte count is at the normal level; ^$^: the odds ratio is calculated per a 1-year increase in age.

**Table 6:** Multiple logistic regression to predict maternal outcomes during COVID-19 infection.

Outcome	Risk Factor	Coefficient	SE	*p*-Value	Adjusted Odds Ratio (aOR)	95% CI for Aor	
						Lower Bound	Upper Bound
Maternal hospitalization ^#^	Severity of COVID infection *	1.352	0.444	0.002	3.9	1.6	9.2
	Dyspnea ^#^	1.937	0.715	0.007	6.9	1.7	28.2
	Lymphocyte count ^&^	1.386	0.823	0.089	3.9	0.8	19.8
Respiratory distress complication ^#^	Age ^$^	0.098	0.043	0.024	1.1	1.01	1.2
	Cough ^#^	1.503	0.746	0.044	4.5	1	19.4
	Dyspnea ^#^	0.764	0.578	0.018	2.1	0.7	6.7
	Chest imaging (Co-RADS infiltration > 50%) ^#^	3.491	0.869	<0.001	32.8	5.9	180.3

“Coefficient” is the estimated regression coefficient. Abbreviations: Co-RADS: COVID-19 reporting and data system. ^#^: the reference category is no; *: the reference category for the severity of COVID infection is a mild state; ^&^: the reference category for lymphocyte count is the normal level; ^$^: the odds ratio is calculated per a 1-year increase in age.

## Data Availability

The data presented in this study are available on request from the corresponding author.
